# The Atlanta Medical College and Its Apologists

**Published:** 1871-07

**Authors:** 


### THE ATLANTA MEDICAL COLLEGE AND ITS APOLOGISTS.

In the June number of the College organ—the Atlanta Medical and Surgical Journal—the editors, with kaleido-copic dexterity, thrown themselves and "the controversy" upon a new line, and take a "new departure." They complacently lull themselves into the belief that the "medical world" will gulph down the assumption that in a recent so-called statement of facts, they have given "*a faithful history*" of the "College trouble," and the *probable* (how modest!) motives which have actuated "the leaders" of the opposition. These leaders—which includes all, save one, who were in attendance at the Augusta meeting of the Medical Association, they declare to be "persecutors"—which they assert they very unpleasantly array before the public "in their true characters." Our Augusta, Savannah and Macon friends should be thankful for the "forbearance" so long exercised—even now, "that patience has ceased to be a virtue." We are told, however, that "to meet the perpetrators of the assaults" and "attempts to traduce the institution" that "physicians" (composed of the Atlanta Academy of Medicine, aspirants to position in the "institution," and its graduates, before and after the "institution" passed under the censure of the profession,) who were opposed (of course!) to the further use, (mark you, *use!*) of the Georgia Medical Association" "attended (yes, every one of them!) the last meeting in Americus, and put the seal of condemnation upon such prostitution (mind you—*prostitution!*) of an Association." Moreover, that these "prostituting parties," "chagrined at failing in their cherished idea of running the Association to the disparagement (save the mark!) of the Atlanta Medical College, the malcontents, consisting chiefly of the nucleus in Atlanta, the Faculty and other friends of the Savannah College, and the friends and expectants, probably, of a contempleted College in Macon (to be sure! the Atlanta Medical College, like the apocryphal tree of Nebuchadnezzer, overshadows the land! and all others in existence, or, prithee, in contemplation, must "blot it out" to succeed,) called a Convention" "in order (alas, poor Yorick!) to perpetuate strife to the detriment of the College in Atlanta."

That is straight talk—it is plain talk; in other words, it is "memorial" talk. Some people ought to have good memories—or at least circumscribe the number of their scribbles. A little over one month ago, a statement of facts, so called, similar to the above, contained language deploring the "memorial" which was lachrymosely mourned over and lamented as having been perpetrated by legal council. Now, however, cannibal like, they devour their own offspring and do not fear to "father the child," which, like Japheth, was so anxiously, in a "statement of facts," "in search of a father,"—notwithstanding, on another page, it is graciously conceded to this child, that "we now condemn ourselves." How far this condemnation goes, may be gathered from the above extracts, which do not abate one jot or tittle of the charges contained in the memorial, but on the contrary, reaffirms and endorses them.

It is asserted again, "that conciliatory acts (meaning, no doubt, Logan's preamble and resolution,) on the part of those traduced, (how and by whom? by the Association at Augusta, Savannah and Macon?) serve only to increase the efforts of their pursuers," and reveals "the motives whieh prompt our persecuters." This blowing of hot and cold in almost the same breath does not establish the "historic verity" of what is complacently styled "a faithful history." To condemn one's self, and then "out Herod Herod" upon the next opportunity, may, possibly, be indicative of character—but can never be regarded as worthy of "historic verity."

We are told—seriously told—that the Convention met and found no College trouble to settle; moreover, that it adjourned "without any action to the disparagement of individuals or institutions." We are thankful for this small crumb of information. It is truly consoling. But our informers, like children, grasp at the moon, and fight at a shadow. It is a pity to disturb such innocent delusions. But the fact is, a "statement of facts" had authoritatively announced to the profession that none but those who were dissatisfied with the action at Americus, or were willing to meet at the beck and call of a faction led by Savannah, would attend the Convention. Veritable history shows, however, that the entire College party (barely 40 in number,) old and young, with the hero of the Americus rescinding feat, as well as almost the entire menbership of a certain Academy—were upon the spot, ready and willing to "undertake" for the "peace and harmony" of the profession, a grand manœuvre, which "packing" so forcibly expresses. When a party wishes to triumph at the polls, success is certain when they can deceive their opponents by saying "no decent man will vote," and then make a "grand rally"—but such victories "settle" nothing save the fact that "deceit and fraud" are the factors of their achievement. Amiability of temper is worthy of all admiration, and we are glad to chronicle that our College friends "indulge no feeling of animosity" towards "the malcontents." We are also thankful for good advice, when it is good. Had the advice which is now so gratuitously offered the profession, been followed by those who now so loudly proffer it, the whole difficulty would have been settled long ago; for our college friends tell us, "we would advise honorable submission to the will of the State Association." But, unfortunately, this State Association is, with them, like the little joker—"now you see it, now you don't." It is a veritable Association, when it suits them and "a meeting assuming to be the Association," when it don't. Yet now, they would have the profession turn aside from its duty by saying, "let the dead bury the dead—follow me." Of course, such advice and such invitations, from such authority, should be followed! We shall see.

After this advice, comes a truth. It is stated that "conciliatory measures and concessions on true points of issue generally lead to reconciliation of honorable parties at variance, but men bent on mischief, making unjust demands, are never appeased by having them granted."

Now the demand made by the Association was right, since they have admitted that their language in the memorial was wrong, and say with a show of feeling, "we condemn ourselves." "Honorable parties at variance" never fear investigation. The College party have uniformly refused, both at Americus and Macon, when by "packing" they had a majority, to submit the questions at issue to a committee. They never did intend to submit to have a settlement by an appeal to the facts alone, in the hands of an impartial committee. They well knew their cause could not stand for a moment, such a test. They knew an honest committee would condemn them, and honorably acquit, justify and applaud the course of those who had simply put themselves in ethical opposition to their violations of medical law. It would have done more —it would have established the principle that a professional man fighting on the line of ethics and principle, must be declared right; and being thus declared, he would then have the endorsement and respect of the profession. This is the true reason why they would not submit to such a course. It would have exalted an enemy, and effectually placed the seal of condemnation upon their own acts.

Individually, and in behalf of the profession assailed, we appeal to facts, and facts only; and therefore invite all to investigate the subject as set forth in a pamphlet, prepared and published by the Fulton Couuty Medical Society, which, unlike a statement of facts," so-called, has been faithfully compiled from the records, as exhibited in the books of the old Board of Trustees. All honorable men will be governed by facts, and facts alone. So far from fearing investigation, we seek and demand it, knowing that truth, in a contest with falsehood, must and will triumph.

---

If the recommendation on the part of a physician, of a nostrum, is sufficient to render him unworthy of professional recognition, why should the slanderer of professional and private character be thought less guilty of violating ethics, when he is the viler of the two. The one is simply dishonest—the other is leprous at heart and worse than an infidel.

